# Relationships between anhedonia, suicidal ideation and suicide attempts in a large sample of physicians

**DOI:** 10.1371/journal.pone.0193619

**Published:** 2018-03-27

**Authors:** Gwenolé Loas, Guillaume Lefebvre, Marianne Rotsaert, Yvon Englert

**Affiliations:** Department of Psychiatry & Laboratory of Psychiatric Research (ULB 266), Cliniques universitaires de Bruxelles, Université Libre de Bruxelles (ULB), Bruxelles, Belgium; Chiba Daigaku, JAPAN

## Abstract

**Background:**

The relationships between anhedonia and suicidal ideation or suicide attempts were explored in a large sample of physicians using the interpersonal psychological theory of suicide. We tested two hypotheses: firstly, that there is a significant relationship between anhedonia and suicidality and, secondly, that anhedonia could mediate the relationships between suicidal ideation or suicide attempts and thwarted belongingness or perceived burdensomeness.

**Methods:**

In a cross-sectional study, 557 physicians filled out several questionnaires measuring suicide risk, depression, using the abridged version of the Beck Depression Inventory (BDI-13), and demographic and job-related information. Ratings of anhedonia, perceived burdensomeness and thwarted belongingness were then extracted from the BDI-13 and the other questionnaires.

**Results:**

Significant relationships were found between anhedonia and suicidal ideation or suicide attempts, even when significant variables or covariates were taken into account and, in particular, depressive symptoms. Mediation analyses showed significant partial or complete mediations, where anhedonia mediated the relationships between suicidal ideation (lifetime or recent) and perceived burdensomeness or thwarted belongingness. For suicide attempts, complete mediation was found only between anhedonia and thwarted belongingness. When the different components of anhedonia were taken into account, dissatisfaction—not the loss of interest or work inhibition—had significant relationships with suicidal ideation, whereas work inhibition had significant relationships with suicide attempts.

**Conclusions:**

Anhedonia and its component of dissatisfaction could be a risk factor for suicidal ideation and could mediate the relationship between suicidal ideation and perceived burdensomeness or thwarted belongingness in physicians. Dissatisfaction, in particular in the workplace, may be explored as a strong predictor of suicidal ideation in physicians.

## Introduction

### Suicide among physicians

High suicide rates among physicians have been reported in various countries. One meta-analysis reported that physicians have a greater risk of death by suicide than the general population [1]. Among potential risk factors for suicide or suicide ideation on physicians, problems at work have been underlined by several studies [2]. Time pressure, work environment, and communication have been described as workplace stressors, predictive of high levels of dissatisfaction at work [2].

The practice of medicine has changed drastically in the past decades with, as a result, a decrease of physicians’ job satisfaction. For example, physicians are now being asked to work in larger groups and be subjected to evaluation relating to the choice of treatments. The development of “evidence based medicine” has led to a decrease of their sense of autonomy. Moreover, economic issues, especially related to liability insurance costs can affect a physician’s perceived job satisfaction. Patients are now also much more informed and tend to defend their right to healthcare. The development of the judicialization of care is a new problem that greatly modifies the general conditions of medical practice [3].

High levels of depression, burn-out or type D personality have been reported in physicians and medical students [4, 5] and these greater levels of personality or mental disorders could be explained, partly, by factors related to the work or university course situation. For example, high levels of dissatisfaction could lead to depression in physicians or medical students [6].

Among the most prominent risks factor of suicide are prior suicide attempts and the presence of a mental disorder, notably mood disorders [7]. High prevalence of suicidal ideations has also been reported in physicians or medical students [8]. Thus, the explorations of factors (e.g. dissatisfaction at work…) that lead to suicide attempts, suicidal ideations or depression are important especially for prevention of suicide.

### Anhedonia and suicide

Anhedonia, a lowered ability to experience pleasure, has been found to be associated with suicidal ideation or suicide attempts in various samples of university students, subjects with depressive spectrum disorders, depressed adolescents, and psychiatric patients [9–12]. The search for a relationship between anhedonia and suicide has, however, provided mixed results [13]. This discrepancy within literature can be explained by, for instance, taking into account the severity and the type of anhedonia. When anhedonia is severe and constitutes an acute dimension, as is the case in depressive disorders, anhedonia is associated with high risk of suicide [14]. When anhedonia is chronic, as is the case in in non-psychiatric subjects or in stable negative schizophrenia subjects, anhedonia is associated with a low level of suicide compared to the studies’ control groups [15, 16]. Recently, anticipatory and consummatory anhedonia have been clearly defined as two separate entities [17]. However, this distinction has not yet been explored in relation to suicidality. Still, several authors [18] previously suggested that recent change in anhedonia and particularly in its social component may be especially predictive of suicidal ideation. Two recent studies have explored this hypothesis.

In the first study, anhedonia was evaluated twice among 1529 psychiatric patients [18], once on admission and once 6 weeks later. Anhedonia was rated using a subscale from the Beck Depression Inventory (BDI-II) which comprised four items (loss of interest, loss of pleasure, loss of interest in sex and loss of energy). Suicidal ideation was rated using the “Suicidal thoughts and wishes” item of the BDI-II. These were separated from a subscale derived from the BDI-II which measured cognitive and affective symptoms of depression. Anhedonia was associated with suicidal ideation at baseline and termination even when the cognitive/affective dimension of depression was controlled for. However, symptom-level analyses showed that loss of interest and loss of pleasure were associated with a higher level of suicidal ideation at baseline and that loss of interest was associated with a higher level of suicidal ideation at termination. Winer et al. [18] suggest that recent change of anhedonia, as rated by the anhedonia subscale of the BDI-II, is associated with suicidal ideation and that loss of interest is the most predictive factor. The authors suggest taking into account the different types of anhedonia when examining suicidal risk.

The second study, by the same team [19] used a sample of 1122 undergraduate students and examined the relationship between recent anhedonia change, suicidal ideation and suicide attempt. Winer et al. [19], used a total of three scales: the Specific Loss of Interest and Pleasure Scale (SLIPS, 11) that rates changes in the ability to take interest or pleasure in social experiences, the Center of Epidemiological Studies Depression Scale (CES-D), and the Suicidal Behaviors Questionnaire-Revised (SBQ-R).

The SLIPS rates both the anticipatory and consummatory components of anhedonia as accurately as the anhedonia subscale from the BDI-II [11]. In a sample of 182 undergraduate students, one study reported significant correlations between the anticipatory or consummatory subscales of the Temporal Experience Pleasure Scale (TEPS) and the SLIPS or the anhedonia subscale of the BDI-II [11]. Another study in various samples or healthy or psychiatric subjects reported significant correlations between the different items of the anhedonia subscale of BDI and the anticipatory or consummatory subscales of the Temporal Experience Pleasure Scale (TEPS) [Loas, personal communication].

Thus, changes in consummatory and anticipatory anhedonia and particularly in its social component were associated with suicidal ideation even when depressive symptoms were controlled for, although the association with suicide attempts was not significant.

The relationships between suicide risk and anhedonia have seldom been studied in physicians or medical students. To the best of our knowledge, only one study has been reported [20].

The study used a sample of 1198 medical students who were participants in a long term study on the precursors of hypertension and coronary disease. Among the 1198 subjects, a subset of 27 subjects were selected by the authors, 9 who died by suicide and two matched controls (i.e. 18) for each participant who died by suicide. The Minnesota-Hartford Personality Assay that scores 20 personality dimensions was rated by a blind reviewer using available records. Anhedonia dimension scores did not significantly differ between the suicide decedents and matched controls. Depression dimension scores were significantly higher in those who died by suicide compared with controls. The main limitations of the study were the use of a non-validated anhedonia scale and the absence of control for depression.

### Interpersonal psychological theory of suicide

The interpersonal psychological theory on suicide (IPT) proposes that three necessary and sufficient conditions lead to suicidal behavior: perceived burdensomeness, thwarted belongingness and the acquired capability for suicide [21]. In this theory, the desire for suicide results from unmet interpersonal needs, an unmet need to belong and an unmet need for social competence leading to perceived burdensomeness. When both thwarted belongingness and perceived burdensomeness are present, desire for suicide appears which, in turn, leads to active suicidal ideation. The acquired capability for suicide is a learned ability to overcome fears associated with pain and death. This ability is acquired through repeated exposure to painful and provocative events. It is necessary for suicidal desire to result in actual serious or lethal suicidal behavior.

It has recently been suggested that the IPT could be applied to physicians and medical trainees [22]. Cornette et al. [22] examined each component of the IPT and found that feelings of burdensomeness could be explained by perceived or actual academic failure, academic burnout, significant financial debt, emotional distress, excessive sense of responsibility for patient problems, and inter-role conflicts. Regarding thwarted belongingness, the authors emphasize that some findings tend to suggest that the medical training environment isn’t favourable to the development of adequate levels of support [23]. Results also indicate that the feeling of interpersonal belongingness is related to a number of sources (e.g. family, friends, co-workers, team or group).

Two recent studies have tested the IPT in physicians. In the first study [24] Fink-Miller had four aims. The first aim was to compare physicians’ scores on IPT components with those reported in other populations. The second aim was to test the hypothesis that thwarted belongingness and perceived burdensomeness predict suicidal ideation. The third aim was to test the hypothesis that thwarted belongingness and perceived burdensomeness interact to predict suicidal ideation. The fourth aim was to test whether thwarted belongingness, perceived burdensomeness and acquired capability interact to predict prior suicide attempts. The author recruited 419 physicians who completed the Interpersonal Needs Questionnaire (INQ) (a 12-item measure which assesses perceived burdensomeness and thwarted belongingness), the Beck Scale for Suicidal Ideation (which assesses suicidal ideation within the past week), and the Acquired Capability for Suicide Scale (ACSS) (which is a five-item measure which assesses fearlessness regarding self-harm). Scores on IPT components were comparable to other groups presenting increased suicidality (i.e. prior attempters, military personnel). Perceived burdensomeness was a significant predictor of suicidal ideation whereas thwarted belongingness predicted prior suicide attempt. Regarding the third and fourth aims, the interactions were not significant. However, acquired capability did not discriminate between prior attempters and non-attempters.

The second study, led by Fink-Miller [25] aimed to examine whether provocative work experiences unique to physicians could predict levels of acquired capability when controlling for gender as well as for painful and provocative experiences outside the work environment. 376 physicians completed the ACSS, the INQ, the pain and provocative events scale, and the life events-medical doctor version. The results showed that the frequency of participation in provocative medical events accurately predicted score on acquired capability, even while controlling for gender and participation in painful and provocative events outside the work environment.

Recent changes in anhedonia and notably in loss of interest in people have been found to be predictive of suicidal ideation, even when accounting for depressive symptoms. Anhedonia, however, was not predictive of suicide attempt when depressive symptoms were controlled for [18]. Winer et al. [18] suggested that the loss of interest in people could be potentially associated with feelings that one does not belong to a group. Anhedonia could lead to social isolation and then to suicidal ideation. Moreover, one study [26] in 167 individuals aged 55 or older recruited from the general USA population reported significant correlations between the SLIPS and the two subscales of the Interpersonal needs Questionnaire rating perceived burdensomeness and thwarted belongingness. The correlations of the SLIPS with the perceived burdensomeness and thwarted belongingness subscales were 0.59 and 0.73 respectively. These three rating scales have significant correlations with the Suicidal Behaviors Questionnaire-revised that rates suicide risk level. Unfortunately, the Suicidal Behaviors Questionnaire-revised does not establish a distinction between suicidal thoughts and attempts.

Taking into account Winer et al.’s suggestion [18] and the results of the Fink-Miller and Golding et al. studies [24–26], the relationships between anhedonia, perceived burdensomeness, thwarted belongingness, suicidal ideations and suicide attempts must be explored and clarified, especially in physicians.

### Anhedonia and interpersonal psychological theory on suicide

Thwarted belongingness comprises the feeling of a lack of reciprocally caring relationships and experience of loneliness. One consequence of thwarted belongingness is social inhibition. A recent study has reported that, in medical students, there was a significant association between anhedonia and social inhibition before and after adjustment for depression [27]. One hypothesis is that thwarted belongingness could lead to anhedonia and then to social inhibition. Social inhibition or social difficulties have been reported in anhedonic subjects. Three studies [review in 27] using college or university students have examined the relationships between anhedonia, rated by the Physical Anhedonia or Social Anhedonia Scales, and expressiveness of emotional communication, social adjustment or social skills in interpersonal situations. When compared to non-anhedonic subjects, anhedonic subjects reported significantly less emotional expressiveness in real-life social situations, had poorer overall social adjustment and were more avoidant, the Cohen *d* effect size for these three studies ranged from .54 to 2.02.

Perceived burdensomeness is characterized notably by self-hate and liability to others. It leads to low self-esteem and feelings of shame or guilt. Introversion could be a cause of these feelings. Numerous studies [28] have reported strong relationship between anhedonia and introversion. Regarding social anhedonia, one study [29] using different rating scales measuring social anhedonia and introversion reported significant correlations between the social anhedonia and the introversion rating scales. In the same study [29], using confirmatory factorial analysis to investigate and clarify the relationship between social anhedonia and introversion, the authors found that the two constructs were separate and that social anhedonia was associated with alexithymia above and beyond any relationship with introversion. One hypothesis is that because social anhedonia is linked to introversion it could lead to perceived burdensomeness. Another hypothesis is that higher levels of alexithymia could lead to social anhedonia and, thus, could hinder the ability to develop interpersonal relationship which would then result in a loss of belongingness.

Thus, the aim of our study was to explore the relationship between recent anhedonia, perceived burdensomeness, thwarted belongingness and lifetime suicidal ideation, recent suicidal ideation, and lifetime suicide attempt. Socio-demographic variables such as age, gender and relationship status were controlled for as were depressive symptoms and the potential effect of medical specialty.

## Methods

An electronic survey was conducted using Google Form Questionnaire software and was sent to 1813 physicians, either currently licensed physicians or residents, i.e. physicians training to be specialists under the supervision of an attending physician, in the city of Brussels, Belgium. Participation was voluntary and the act of logging on served as informed consent. Among the 1813 physicians there were 1397 physicians working in hospitals and 416 in medical offices. The Ethics board of the Erasme Hospital examined and approved the project (reference P2014/459, agreement date 12/15/2014). The group of 1813 physicians consisted of 416 self-employed general practitioners and 1397 physicians employed in five hospitals located in Brussels (i.e. Bordet, Brugmann, Huderf, Erasme and Saint Pierre hospitals). The five hospitals were related to the network of the Université Libre de Bruxelles (ULB). The survey was conducted from January to April 2015.

The questionnaire took 15 to 30 minutes to complete and consisted of 72 questions. 59 questions measured work-related factors, health-related behavior, psychological distress and demographic factors. The remaining 13 items were taken from the abridged form of the Beck depression inventory (BDI-13).

### Measures

#### Independent variables

In this study we did not use measures specifically designed to assess the IPT such as the Interpersonal Needs Questionnaire (INQ, [30]). Instead, we used various items from our questionnaire to create proxy scales of belongingness and burdensomeness. This approach has several limitations and we took particular effort to validate the constructs. Firstly, the three first authors reviewed the questionnaire and, independently, selected items that they thought were strongly related to the constructs. Secondly, the list of the items was then reviewed by the authors, retaining only the items that proved to be consistent across lists. Thirdly, discrepant items were discussed and a decision was made by consensus about whether to retain or exclude these items. Fourthly, Cronbach’s alpha coefficients were calculated to determine reliability of the proxy scales.

The Perceived burdensomeness and thwarted belongingness scale contains 14 items (7 for each subscale). The items rated the current state or the last two weeks but some items specify both current and past 5 years. Globally the time frame is the current state. The alpha Cronbach was 0.75 with a mean inter-item correlation of 0.18. The perceived burdensomeness subscale (PB) comprised seven items (see Table 1). The alpha Cronbach was 0.57 with a mean inter-item correlation of 0.17. The correlations between each item and the total score were significant and ranged from 0.31 to 0.53 with a mean of 0.44. The seven items of the PB correlated weakly with the thwarted belongingness scale (TB) score. The values ranged from 0.05 (not significant) to 0.3 with a mean value of 0.18. The TB comprised seven items (see Table 1). The alpha Cronbach was 0.64 with a mean inter-item correlation of 0.21. The correlations between each item and the total score were significant and ranged from 0.33 to 0.6 with a mean of 0.49. The seven items of the TB correlated weakly with the PB score; the values ranged from 0.07 (not significant) to 0.33 with a mean value of 0.19. TB and PB were significantly correlated (Spearman’s rho = 0.38, p <0.05).

**Table 1:** Items from the derived scales of the interpersonal psychological theory of suicide theory constructs.

**Perceived burdensomeness scale (PB)**	Yes	No
Do you have a medical condition (chronic pain, disabling illness…)?	ϒ	ϒ
Are you currently encountering financial problems?	ϒ	ϒ
Are you aware of any organisation that would be able to help you if you were faced with a personal problem? If so, please let us know which one	ϒ	ϒ
In any given situation, do you easily feel that you’re to blame?	ϒ	ϒ
Would you ask for specialized help if you were faced with a mental illness yourself?	ϒ	ϒ
In relation to general guidelines, do you often feel limited in how far you can help patients because of limited resources available to you?	ϒ	ϒ
Would you feel humiliated if you had to ask for help?	ϒ	ϒ
**Thwarted belongingness scale (TB)**	Yes	No
Are you currently encountering relational problems (dispute with a current or former partner or with anyone else, bereavement, has anyone–friend or family- died or committed suicide in the past five years)?	ϒ	ϒ
Have you had to deal with an unsettling event (for instance, a break-up or a dispute with someone close) in the past two weeks?	ϒ	ϒ
Do you feel limited because of life choices?	ϒ	ϒ
Have you noticed a rise in the amount of lawsuits in the medical profession?	ϒ	ϒ
Do you feel that you are competing against your colleagues?	ϒ	ϒ
Would you worry about confidentiality not being observed if you were to turn to a colleague when faced with a personal crisis?	ϒ	ϒ
Would you worry about being seen by someone you know in the waiting room if you were to consult a colleague?	ϒ	ϒ

Anhedonia (ANH-BDI-13): We used the short form of the Beck Depression Inventory (BDI-13) that contains 13 of the 21 items of the original scale introduced in 1961. The items of the BDI-13 and BDI were numbered using capital letters. The BDI-13 comprised 13 items from item # A “Mood” to item # M “Loss of appetite” and the BDI comprised 21 items from item # A “Mood” to item # U “Loss of libido”. The capital letters assigned to items from the BDI-13 were in no way linked to the capital letters assigned to items from and the BDI (e.g. the item rating social withdrawal is numbered # H in the BDI-13 and # L in the BDI). The French version of the BDI-13 has satisfactory psychometric properties. In the present study, the alpha Cronbach was 0.91 with a mean inter-item correlation of 0.6.

In 1996, the BDI-II has been developed in order to adhere more closely to the diagnostic criteria for major depressive episode in the DSM-IV. Contrary to the original BDI and BDI-13, the items of the BDI-II were numbered using Arabic numerals from # 1 “Sadness” to # 21 “Loss of interest in sex”. The item “work inhibition” was dropped from the BDI and replaced by a “loss of energy” item in the BDI-II.

To facilitate the comprehension we used only the Arabic numerals for the items.

Anhedonia was rated using the anhedonia subscale of the BDI as used by Joiner et al. [31]. The anhedonia subscale contains three items (item # 4: lack of satisfaction, item # 12: social withdrawal or loss of interest and item # 21: loss of interest in sex). Other authors [18, 32] included loss of energy (item # 15) in the anhedonia subscale whereas Joiner [31] or Leventhal [33] did not. All of these authors used either the original BDI [31], or the revised version (BDI-II) [18, 32, 33].

Item # 4 rates a lack of satisfaction in the BDI and loss of pleasure in the BDI-II. Item #12 rates social withdrawal in the BDI and loss of interest in the BDI-II. The phrasing of item # 12 in both cases is quasi-identical: in the BDI, the item rates the loss of interest in other people, whereas, in the BDI-II it rates a loss of interest in other people or activities. Thus, in the BDI item # 12 is centered only on people. Item # 21 (Loss of libido), however, is very similar in the original and revised versions of the BDI.

The short form of the BDI includes 3 items of the original version that can be used to rate anhedonia: item # 4 (Lack of satisfaction), item # 12 (Social withdrawal or loss of interest) and item # 15 (Work inhibition).

Joiner et al. [31] reported low reliability (alpha coefficient of 0.57) of the anhedonia subscale in their study of 102 patients presenting either major depression or schizophrenia. However, in two other samples the values of the alpha coefficients were higher (0.69 and 0.73). In the BDI-13, loss of interest in sex was not included. Instead, three other BDI items were used: Lack of satisfaction (LS-BDI-13) (ranging from 0: “I am not particularly dissatisfied” to 3: “I am dissatisfied with everything”); Loss of interest (LI-BDI-13) (ranging from 0: “I have not lost interest in other people” to 3: ”I have lost all interest in other people and don’t care about them at all.”); Work inhibition (WI-BDI-13) (ranging from 0: “I can work as well as before” to 3: “I can’t do any work at all”). The alpha Cronbach for this three-item scale was 0.59 with a mean inter-item correlation of 0.33.

The BDI items are rated using statements that best describe how the subjects have felt during the previous 2 weeks. Thus, the BDI evaluates recent changes or recent depressive symptoms. As the SLIPS [11], the anhedonia subscale of the BDI rates both the recent (or change) anticipatory and consummatory components of anhedonia.

Cognitive/affective symptoms of depression (CA-BDI-13) were assessed using the summation of four items of the BDI-13: sense of failure, guilt, self-hate and self-body image. The alpha Cronbach was 0.71 with a mean inter-item correlation of 0.38. Winer et al. [18] used a 6-item subscale of the BDI-II to measure the cognitive/affective symptoms of depression. Four of these six items were identical to the abridged version of the BDI and were chosen for the present study, thus allowing a comparison between our results and the Winer et al. study [18].

Clinical specialties: The following specialties were taken into account: paediatrics, obstetrics and gynaecology, clinical laboratory specialties, anaesthesiology and intensive care medicine, surgery (including ophthalmologists and otolaryngologists), radiology (including radiation therapy and nuclear medicine), internal medicine (including rheumatology, oncology, hematology, nephrology, geriatry, pulmonary medicine, cardiology, endocrinology, gastroenterology) psychiatry and child psychiatry, and general practice. Previous studies on stress, burnout or suicide in physicians used similar classification of specialties [34, 35]. A marked difference in prevalence of suicide has also been observed between medical specialties, with psychiatrists and anaesthesiologists being at greater risk of suicide compared to other physicians. Paediatricians and radiologists appear to have the lowest risk [36].

#### Dependent variables

There were three dependent variables: Lifetime suicidal ideation (lf-SI) and lifetime suicide attempts (lf-SA) and recent suicidal ideation (SI). Lifetime suicidal ideation (lf-SI) and lifetime suicide attempts (lf-SA) were rated using just two questions of the questionnaire (Have you had any suicidal ideas? Did you make a suicide attempt?). For recent suicidal ideation (SI), the “Suicidal thoughts or wishes” item of the BDI-13 was used in which 0 is “I don't have any thoughts of harming myself” and 3 is “I would like to kill myself if I could”. The responses for each of the dependent variables were dichotomized with a score of 0 (i.e. no lifetime suicidal ideation, no lifetime suicide attempt, response 0 to the “Suicidal thought and wishes” item of the BDI-13) and 1 (i.e. lifetime suicidal ideation, at least one suicide attempt, response higher than 0 to the “Suicidal thought and wishes” item of the BDI-13).

#### Covariates

Age, gender and relationship status (single or not) were the three variables taken into account.

### Statistical analyses

Firstly, bivariate statistical analyses were performed using each of the dependent variables and covariates (age, gender, relationship status) and the independent variables (PB, TB, ANH-BDI-13, LS-BDI-13, LI-BDI-13, WI-BDI-13, CA-BDI-13, clinical specialties). Chi-square analyses with Yates correction or Mann Whitney U tests were carried out. The level of significance was p < 0.05. No Bonferroni correction was applied as the significant covariates or independent variables were introduced in the multivariate analyses.

Secondly, logistic regressions were performed using lifetime suicidal ideation, lifetime suicide attempts or recent suicidal ideation as dependent variables. Then, covariates and independent variables which had been found to be significant in the bivariate analyses were used.

Thirdly, mediation analyses were performed. A formal mediation analysis was carried out in order to determine if anhedonia mediates the relationship between perceived burdensomeness or thwarted belongingness and lifetime suicidal ideation, lifetime suicide attempt or recent suicidal ideation. Using the procedure proposed by Baron and Kenny [37], a series of hierarchical regressions were performed as well as a Sobel test.

According to Baron and Kenny [37] mediation is proved when the following conditions are observed: (1) the independent variable (perceived burdensomeness or thwarted belongingness) affects the mediator (anhedonia); (2) the independent variable (perceived burdensomeness or thwarted belongingness) affects the dependent variable (lifetime suicidal ideation, lifetime suicide attempt or recent suicidal ideation); (3) the mediator (anhedonia) affects the dependent variable (lifetime suicidal ideation, lifetime suicide attempt or recent suicidal ideation) when the independent variable (perceived burdensomeness or thwarted belongingness) is controlled for and (4) complete mediation is confirmed when the relationship between the independent variable (perceived burdensomeness or thwarted belongingness) and dependent variable (lifetime suicidal ideation, lifetime suicide attempt or recent suicidal ideation) become non-significant after the effect of the mediator (anhedonia) is controlled for. If the conditions 1–3 are met, then there is partial mediation.

## Results

Among the 1813 physicians, 557 agreed to participate (31%). There were 557 physicians (223 males, 334 females) with a mean age of 39.21 years (*SD* = 12.42; range: 27–68). The different clinical specialties were: paediatrics (*N* = 55), obstetrics and gynaecology (*N* = 32), clinical laboratory specialties (*N* = 31), anaesthesiology and intensive care medicine (*N* = 58), surgery (*N* = 83), radiology (*N* = 37), internal medicine (*N* = 152), psychiatry and child psychiatry (*N* = 31), general practice (*N* = 78). There were 366 licensed physicians and 191 residents. There were 479 physicians working in hospitals and 78 working in private offices.

### Bivariate analyses (see Table 2)

#### Lifetime suicidal ideations (lf-SI)

133 physicians (23.9%) had lf-SI. Regarding the covariates, the prevalence of women (68.4%) was significantly higher in physicians with lf-SI than those found (57.3%) in physicians without lf-SI (*χ2* = 5.2, *df* = 1, *p* = 0.022) and physicians with lf-SI were significantly less likely to be in a relationship (29.3% were single) than the physicians without lf-SI (16.7% were single) (*χ2* = 10.1, *df* = 1, *p* = 0.001). No significant difference was reported for age. Regarding the independent variables, except for the independent variable “clinical specialties”, significant differences were found with higher scores on the various scales (see Table 2).

**Table 2:** Bivariate analyses of demographic and psychometrical variables between physicians having or not life-time (lf) suicidal ideation, life-time (lf) suicide attempts or recent suicidal ideation.

	Without lf-Suicidal Ideations	lf-Suicidal Ideations			Without lf-Suicide attempts	lf-Suicide attempts			Without Suicidal Ideations	Suicidal Ideations		
	(n = 424)	(n = 133)			(n = 543)	(n = 14)			(n = 533)	(n = 24)		
	N (%)	N (%)	χ2	*p*	N (%)	N (%)	χ2	p	N (%)	N (%)	χ2	p
Gender Female/Male	**Female:243 (57.3%)** **Male:181 (42.7%)**	**Female:91(68.4%)** **Male:42(31.6%)**	**5.2**	**0.022**	Female:325 (59.8%) Male:218 (40.2%)	Female:9 (64.3%) Male:5 (35.7%)	0.03	0.85	**Female:313 (58.7%)** **Male:220 (41.3%)**	**Female:21 (87.5%)** **Male:3 (12.5%)**	**6.77**	**0.009**
Relationship status Not-Single/ Single	**N-Single:353 (83.3%)** **Single:71** **(16.7%)**	**N-Single:94 (70.7%)** **Single:39 (29.3%)**	**10.1**	**0.001**	N-Single:437 (80.5%) Single:106 (19.5%)	N-Single:10 (71.4%) Single:4 (28.6%)	0.25	0.62	N-Single:430 (80.7%) Sigle:130 (19.3%)	N-Single:17 (70.8%) Single:7 (29.2%)	0.85	0.36
	Mean (SD)	Mean (SD)	*U (z)*	*p*	Mean (SD)	Mean (SD)	U (z)	p	Mean (SD)	Mean (SD)	*U (z)*	*p*
Age	39.13 (12.41)	39.44(12.48)	0.09	0.93	39.07 (12.39)	44.46 (12.79)	1.74	0.08	39.16 (12.41)	40.21 (12.85)	0.33	0.743
**Perceived burdensomeness scale**	**1.94 (1.14)**	**2.74 (1.43)**	**5.62**	**0.001**	2.11 (1.26)	2.79 (1.37)	1.79	0.07	**2.08 (1.25)**	**3.08 (1.25)**	**3.72**	**0.001**
**Thwarted belongingness scale**	**1.89 (1.41)**	**2.98 (1.71)**	**6.47**	**0.001**	**2.12 (1.55)**	**3.5 (1.4)**	**3.21**	**0.001**	**2.09 (1.54)**	**3.46 (1.5)**	**3.99**	**0.001**
Anhedonia subscale-BDI-13	**0.84 (1)**	**1.68 (1.26)**	**7.03**	**0.001**	**1.01 (1.11)**	**2.14 (1.17)**	**3.51**	**0.001**	**0.98 (1.08)**	**2.42 (1.18)**	**5.44**	**0.0001**
Lack Satisfaction-BDI-13	**0.28 (0.45)**	**0.62 (0.54)**	**5.66**	**0.003**	**0.35 (0.49)**	**0.71 (0.61)**	**2.04**	**0.041**	**0.33 (0.48)**	**0.96 (0.55)**	**4.49**	**0.001**
Lack of Interest-BDI-13	**0.23 (0.42)**	**0.38 (0.55)**	**2.25**	**0.024**	0.27 (0.46)	0.29 (0.47)	0.15	0.87	**0.26 (0.45)**	**0.5 (0.51)**	**2.05**	**0.004**
Work Inhibition-BDI-13	**0.33 (0.51)**	**0.67 (0.62)**	**5.89**	**0.001**	**0.39 (0.54)**	**1.14 (0.66)**	**4.31**	**0.001**	**0.39 (0.54)**	**0.96 (0.62)**	**4.58**	**0.0001**
Cognitive/Affective-BDI-13	**0.64 (1.15)**	**2.12 (2.19)**	**8.53**	**0.001**	**0.95 (1.53)**	**2.64 (2.76)**	**3.28**	**0.001**	**0.87 (1.44)**	**3.62 (2.43)**	**6.49**	**0.001**

(Perceived burdensomeness (PB); Thwarted belongingness (TB); Anhedonia subscale of the Abridged version of the Beck Depression Inventory (ANH-BDI-13); Lack of satisfaction item of the BDI-13 (LS-BDI-13); Lack of interest of the BDI-13 (LI-BDI-13); Work inhibition of the BDI-13 (WI-BDI-13); Cognitive/affective symptoms of depression (CA-BDI-13); Comparison: (1) between physicians with or without lf suicidal ideation; (2) between physicians with or without lf suicide attempt; (3) between physicians with or without recent suicidal ideation using Chi square (χ2) or Mann-Whitney U test tests. In bold face p < 0.05).

#### Lifetime suicide attempts (lf-SA)

14 (2.5%) physicians had lf-SA. Regarding the covariates, there were no significant differences. For the independent variables, except for the independent variable “clinical specialties”, perceived burdensomeness and Loss of interest -BDI-13, significant differences were found (see Table 2).

#### Recent suicidal ideations (SI)

24 (4.3%) physicians had SI. Regarding the covariates, the prevalence of women (87.5%) was significantly higher in physicians with SI than those found (58.7%) in physicians without SI (*χ2* = 6.77, *df* = 1, *p* = 0.009). Except for the independent variable “clinical specialties”, every independent variable turned out to be significantly different between the physicians who had had recent suicidal ideations and physicians who hadn’t.

### Multivariate analyses (see Table 3)

Six logistic regression analyses were performed using, firstly, lifetime suicidal ideations, lifetime suicide attempts and recent suicidal ideations as dependent variables and, secondly, the socio-demographic (covariates) and independent variables that had been found to be significant in the bivariate analyses. For each dependent variable there were two logistic regressions taken into account, either a model using the total Anhedonia-BDI-13 (ANH-BDI-13) score, or a model using Lack of Satisfaction (LS-BDI-13), Loss of Interest (LI-BDI-13), and work inhibition (WI-BDI-13) scores separately. Taking into account that several independent variables are correlated and to avoid multicollinearity, ridge regressions were also used.

**Table 3:** Multivariate analyses of demographic and psychometrical variables between physicians having or not life-time (lf) suicidal ideation, life-time (lf) suicide attempts or recent suicidal ideation.

Predictors	Lifetime suicidal ideation				Lifetime suicide attempts				Recent suicidal ideations			
	*Wald’s*_*X*_^*2*^	*OR*	*95% CI*	*p*	*Wald’s*_*X*_^*2*^	*OR*	*95% CI*	*p*	*Wald’s*_*X*_^*2*^	*OR*	*95% CI*	*p*
Gender	0.29	0.88	0.55–1.40	0.59	/				0.92	0.54	0.15–1.92	0.34
Relationship status	**4.28**	**1.7**	**1.03–2.85**	**0.04**	/				/			
Anhedonia BDI-13	**3.87**	**1.25**	**1–1.56**	**0.05**	2.46	1.52	0.9–2.56	0.12	**4.17**	**1.57**	**1.01–2.43**	**0.04**
Cognitive/Affective-BDI-13	**17.97**	**1.4**	**1.2–1.64**	**0.001**	1.3	1.18	0.89–1.56	0.25	**10.84**	**1.46**	**1.17–1.84**	**0.001**
Perceived Burdensomeness scale	**3.94**	**1.21**	**1–1.46**	**0.05**	/				0.7	1.17	0.81–1.67	0.4
Thwarted Belongingness scale	**4.1**	**1.17**	**1–1.37**	**0.04**	2.42	1.34	0.93–1.93	0.12	0.45	1.11	0.81–1.52	0.5
Gender	0.21	0.9	0.56–1.43	0.65	/				0.00	0.97	0.34–2.76	0.95
Relationship status	3.55	1.64	0.98–2.75	0.06	/				/			
Lack Satisfaction-BDI-13	**4.5**	**1.71**	**1.2–2.61**	**0.034**	0.01	1.07	0.3–3.81	0.92	**7.71**	**4.21**	**1.52–12.8**	**0.005**
Loss of Interest-BDI-13	0.009	0.6	1.59–0.95	0.92	/				1.15	1.59	0.68–3.7	0.28
Work Inhibition-BDI-13	0.99	1.25	0.81–1.93	0.32	**8.68**	**4.83**	**1.69–13.78**	**0.003**	1.54	1.69	0.74–3.9	0.21
Cognitive/Affective-BDI-13	**16.20**	**1.38**	**1.18–1.62**	**0.001**	0.26	1.08	0.8–1.46	0.61	**14.92**	**1.6**	**1.26–2.03**	**0.001**
Perceived Burdensomeness scale	**3.97**	**1.21**	**1–1.46**	**0.046**	/				2.49	1.33	0.93–1.9	0.11
Thwarted Belongingness scale	**3.86**	**1.17**	**1–1.37**	**0.049**	2.05	1.31	0.9–1.92	0.15	0.07	1.04	0.76–1.44	0.79

(In bold face p < 0.05).

For lifetime suicidal ideations, there were two significant covariates (gender and relationship status) and seven significant independent variables (Anhedonia-BDI-13 (ANH-BDI-13), Lack of Satisfaction-BDI-13 (LS-BDI-13), Loss of Interest-BDI-13 (LI-BDI-13), Work Inhibition-BDI-13 (WI-BDI-13), Cognitive/affective symptoms of depression (CA-BDI-13), perceived burdensomeness (PB) and thwarted belongingness (TB)).

Firstly, using ANH-BDI-13, the global test was significant (*χ2* = 105, 13, *df* = 6, *p* = 0.001). Relationship status was significant (Wald chi-square = 4.28, *p* = 0.04; *OR* = 1.7; *95% CI* = 1.03–2.85) as was CA-BDI-13 (Wald chi-square = 17.97, p = 0.001; OR = 1.4; 95%CI = 1.2–1.64), PB (Wald chi-square = 3.94, *p* = 0.05; *OR* = 1.21; *95%CI* = 1–1.46), TB (Wald chi-square = 4.1, *p* = 0.04; *OR* = 1.17; *95%CI* = 1–1.37) and ANH-BDI-13 (Wald chi-square = 3.87, *p* = 0.05; *OR* = 1.25; *95%CI* = 1–1.56)). Ridge regressions produced similar results. The proportions of variance explained by the predictors are: 14.3% (CA-BDI-13); 2.24% (TB); 1.1% (ANH-BDI-13); 0.7% (PB) and 0.6% (Relationship status).

Secondly, when LS, LI and WI-BDI-13 were used instead of ANH-BDI-13, the global test was significant (*χ2* = 106, 91, *df* = 8, *p* = 0.001). CA-BDI-13 was significant (Wald chi-square = 16.2, *p* = 0.005; *OR* = 1.38; *95%CI* = 1.18–1.62), as was PB (Wald chi-square = 3.97, p = 0.046; *OR* = 1.21; *95%CI* = 1–1.46), and TB (Wald chi-square = 3.86, *p* = 0.049; *OR* = 1.17; *95%CI* = 1–1.37). Ridge regressions produced different results with four significant predictors, LS-BDI-13 becoming significant. The proportions of variance explained by the predictors are: 14.3% (CA-BDI-13); 2.24% (TB); 1.2% (LS-BDI-13); 0.8% (PB) and 0.5% (Relationship status).

For lifetime suicide attempts, there were five independent variables (Anhedonia-BDI-13 (ANH-BDI-13), Lack of Satisfaction-BDI-13 (LS-BDI-13), Work Inhibition-BDI-13 (WI-BDI-13), Cognitive/affective symptoms of depression (CA-BDI-13) and thwarted belongingness (TB)). Firstly, using ANH-BDI-13, the global test was significant (*χ2* = 16.4, *df* = 3, *p* = 0.001) and there were no significant predictors. Secondly, when LS-BDI-13 and WI-BDI-13 were used instead of ANH-BDI-13, the global test was significant (*χ2* = 23.67, *df* = 4, *p* = 0.001) and only WI-BDI-13 was significant (Wald chi-square = 8.68, *p* = 0.003; *OR* = 4.83; *95% CI* = 1.69–13.78). Ridge regressions produced similar results. The proportion of variance explained by WI-BDI-13 was 4.1%.

For recent suicidal ideations, there was one significant covariate (gender) and seven independent variables (Anhedonia-BDI-13 (ANH-BDI-13), Lack of Satisfaction-BDI-13 (LS-BDI-13), Loss of Interest-BDI-13 (LI-BDI-13), Work Inhibition-BDI-13 (WI-BDI-13), Cognitive/affective symptoms of depression (CA-BDI-13), thwarted belongingness (TB), perceived burdensomeness (PB)). Firstly, using ANH-BDI-13, the global test was significant (*χ2* = 52.05, *df* = 5, *p* = 0.001). CA-BDI-13 was significant (Wald chi-square = 10.84, *p* = 0.001; *OR* = 1.46; *95%CI* = 1.17–1.84) as was ANH-BDI-13 (Wald chi-square = 4.17, *p* = 0.04; *OR* = 1.57; *95%CI* = 1–2.43). The proportion of variance explained by CA-BDI-13 and ANH-BDI-13 were respectively 11.18% and 1%. Ridge regression produced similar results. Secondly, when LS-BDI-13, LI-BDI-13 and WI-BDI-13 were used instead of ANH-BDI-13, the global test was significant (*χ2* = 42.76, *df* = 7, *p* = 0.001). Only CA-BDI-13 was significant (Wald chi-square = 14.92, *p* = 0.001; *OR* = 1.6; *95% CI =* 1.26–2.03). Ridge regression reported that CA-BDI-13 and LS-BDI-13 were significant. CA-BDI-13 and LS-BDI-13 explained 11.2% and 1.22% of the variance, respectively.

### Mediation analyses

Several mediation analyses were performed using perceived burdensomeness (PB) or thwarted belongingness (TB) as independent variables, Anhedonia-BDI-13 (Anh-BDI-13), Lack of satisfaction BDI-13 (LS-BDI-13), Loss of interest BDI (LI-BDI-13) or work inhibition BDI-13 (WI-BDI-13) as mediators, and life suicidal ideation, lifetime suicide attempts or recent suicidal ideations as dependent variables.

**Fig 1:**
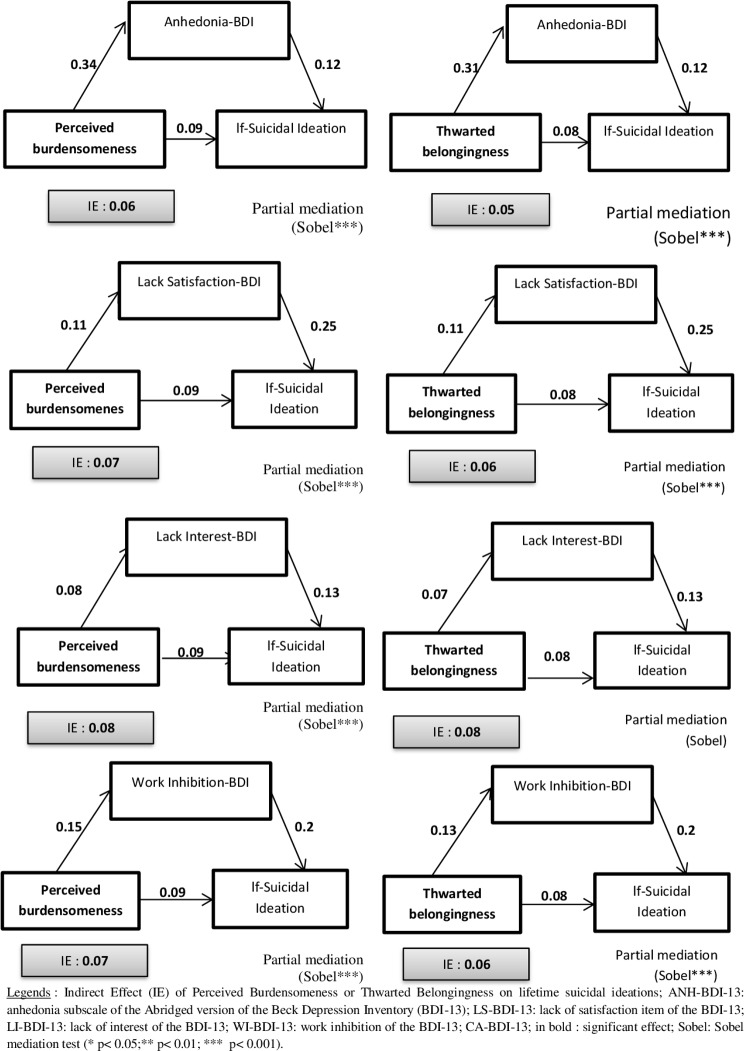
Mediation analyses for lifetime (lf) suicidal ideations.

**Lifetime suicidal ideations (see Fig 1)**

Perceived Burdensomeness (PB) as independent variable

Anhedonia-BDI-13 as mediator

Perceived Burdensomeness predicted Anh-BDI-13 (*B* (non-standardized) = 0.34, *t* (555) = 9.7, *p* = 0.001) hereby indicating that condition 1 for mediation was met. Perceived Burdensomeness was a significant predictor of lifetime suicidal ideation at step 1 (*B* = .09, *t* (555) = 6.62, *p* = 0.001) hence showing that condition 2 was met. When Anh-BDI-13 was entered in the equation at step 2 it significantly predicted lifetime suicidal ideation (*B* = .09, *t* (554) = 5.81 *p* = 0.001) and reduced the beta weight for Perceived Burdensomeness (*B* = .06, *t* (554) = 4.1, *p* = 0.005), but not to a level of non-significance. Thus, condition 4 having not been met, there was only partial mediation. The Sobel test was significant (z = 4.98, *p* = 0.001).

Lack of Satisfaction-BDI-13 as mediator

Partial mediation was found with a significant Sobel’s test (*z* = 4.456, *p* = 0.001).

Loss of Interest-BDI-13 as mediator

Partial mediation was found with a significant Sobel test (*z* = 2.81, *p* = 0.005).

Work Inhibition-BDI-13 as mediator

Partial mediation was found with a significant Sobel test (*z* = 6.46, *p* = 0.001).

Thwarted Belongingness (TB) as independent variable

Anhedonia-BDI-13 as mediator

Thwarted Belongingness predicted Anh-BDI-13 (*B* = .31, *t* (555) = 11.2, *p* = 0.001) hereby indicating that condition 1 for mediation was met. Thwarted Belongingness was a significant predictor of lifetime suicidal ideation at step 1 (*B* = .08, *t* (555) = 7.4, *p* = 0.001) hence showing that condition 2 was met. When Anh-BDI-13 was entered in the equation at step 2 it significantly predicted lifetime suicidal ideation (*B* = 0.08, *t* (554) = 5.25 *p* = 0.001) and reduced the beta weight for Thwarted Belongingness (*B* = .05, *t* (554) = 4.6, *p* = 0.001), but not to a level of non-significance. Thus, condition 4 not having been met, there was only partial mediation. The Sobel test was significant (*z* = 4.75, *p* = 0.001).

Lack of Satisfaction-BDI-13 as mediator

Partial mediation was found with a significant Sobel’s test (*z* = 4.41, *p* = 0.001).

Loss of Interest-BDI-13 as mediator

Partial mediation was found with a non-significant Sobel’s test (*z* = 1.74, *p* = 0.082).

Work Inhibition-BDI-13 as mediator

Partial mediation was found with a non-significant Sobel’s test (*z* = 4.74, *p* = 0.001).

**Fig 2:**
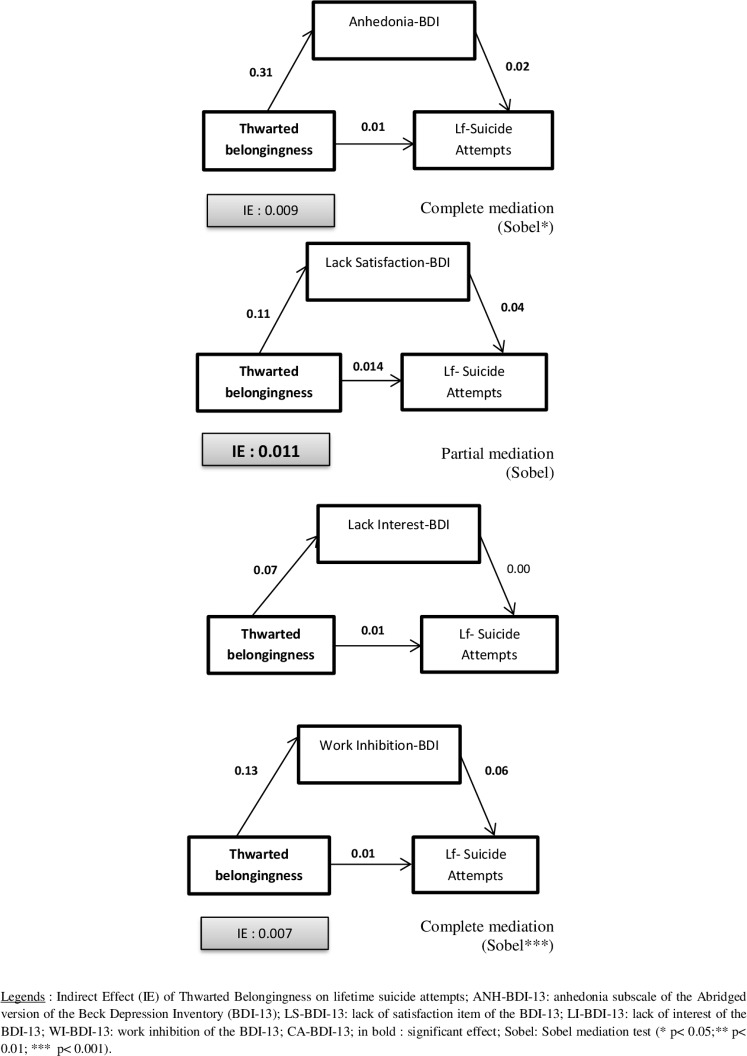
Mediation analyses for lifetime suicide attempts.

**Lifetime suicide attempts (see Fig 2)**

Thwarted Belongingness (TB) as an independent variable

Anhedonia-BDI-13 as mediator

Thwarted Belongingness predicted ANH-BDI-13 (*B* = .31, *t* (555) = 11.2, *p* = 0.001), hereby indicating that condition 1 for mediation was met. Thwarted Belongingness was a significant predictor of lifetime suicide attempt at step 1 (*B* = .01, *t* (555) = 3.3, *p* = 0.001) hence showing that condition 2 was met. When ANH-BDI-13 was entered in the equation at step 2 it significantly predicted lifetime suicidal attempt (*B* = .017, *t* (554) = 2.58 *p* = 0.01) and reduced the beta weight for Thwarted Belongingness (*B* = .009, *t* (554) = 1.89, *p* = 0.059) to a level of non-significance. Thus condition 4 having been met, there was complete mediation. The Sobel test was significant (*z* = 2.51, p = 0.012).

Lack of Satisfaction-BDI-13 as mediator

Partial mediation was found with a non-significant Sobel’s test (*z* = 1.66, *p* = 0.1).

Loss of Interest-BDI-13 as mediator

The regression between LI-BDI-ANH and lifetime suicide attempts was not significant and thus the mediation analysis was not taken into account.

Work Inhibition-BDI-13 as mediator

Complete mediation was found with significant Sobel’s test (*z* = 3.8, *p* = 0.001).

Perceived Burdensomeness (PB) as an independent variable

Perceived Burdensomeness was not a significant predictor of lifetime SA, and thus mediation was not explored.

**Fig 3:**
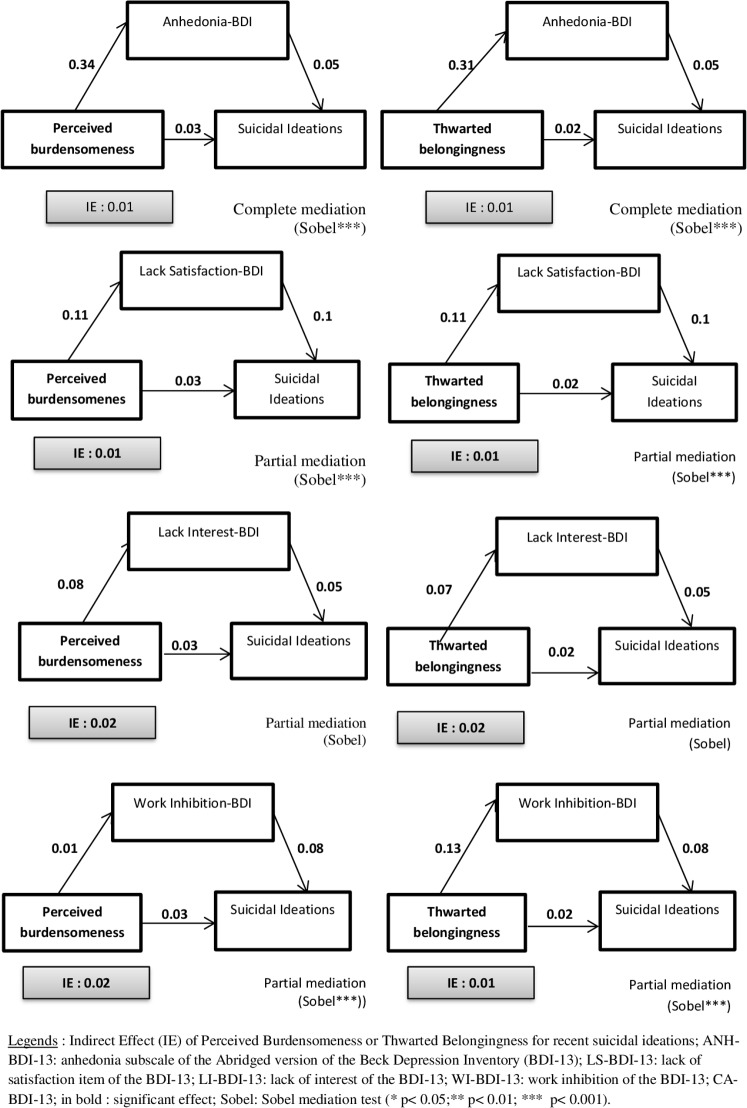
Mediation analyses for recent suicidal ideations (SI).

**Recent suicidal ideations (see Fig 3)**

Perceived Burdensomeness (PB) as independent variable

Anhedonia-BDI-13 as mediator

Perceived Burdensomeness predicted ANH-BDI-13 (*B* = .34, *t* (555) = 9.7, *p* = 0.001) hereby indicating that condition 1 for mediation was met. Perceived Burdensomeness was a significant predictor of recent suicidal ideation at step 1 (*B* = .03, *t* (555) = 3.84, *p* = 0.001) hence showing that condition 2 was met. When ANH-BDI-13 was entered in the equation at step 2 it significantly predicted recent suicidal ideation (*B* = .04, *t* (554) = 5.23 *p* = 0.001) and reduced the beta weight for Perceived Burdensomeness (*B* = .011, *t* (554) = 1.65, *p* = 0.1), to a level of non-significance. Thus condition 4 having been met, there was full mediation. The Sobel test was significant (*z* = 4.6, *p* = 0.001).

Lack of Satisfaction-BDI-13 as mediator

Partial mediation was found with a significant Sobel’s test (*z* = 4.25, *p* = 0.001).

Loss of Interest-BDI-13 as mediator

Partial mediation was found with a non-significant Sobel’s test (*z* = 1.7, *p* = 0.09).

Work Inhibition-BDI-13 as mediator

Partial mediation was found with a significant Sobel’s test (*z* = 3.59, *p* = 0.003).

Thwarted Belongingness (TB) as independent variable

Anhedonia-BDI-13 as mediator

Thwarted Belongingness predicted ANH-BDI-13 (*B* = .31, *t* (555) = 11.2, *p* = 0.001) hereby indicating that condition 1 for mediation was met. Thwarted Belongingness was a significant predictor of recent suicidal ideation at step 1 (*B* = .02, *t* (555) = 4.26, *p* = 0.001) hence showing that condition 2 was met. When ANH-BDI-13 was entered in the equation at step 2 it predicted significant recent suicidal ideation (*B* = .04, *t* (554) = 4.95 *p* = 0.001) and reduced the beta weight for Thwarted Belongingness (*B* = .011, *t* (554) = 1.81, *p* = 0.07), to a level of non-significance. Thus, condition 4 having been met, there was full mediation. The Sobel test was significant (z = 4.55, *p* = 0.001).

Lack of Satisfaction-BDI-13 as mediator

Partial mediation was found with a significant Sobel’s test (z = 4.33, *p* = 0.001).

Loss of Interest-BDI-13 as mediator

Partial mediation was found with a non-significant Sobel’s test (*z* = 1.57, *p* = 0.12).

Work Inhibition-BDI-13 as mediator

Partial mediation was found with a significant Sobel’s test (*z* = 3.46, *p* = 0.005).

## Discussion

The aim of the present study was to examine the relationship between recent anhedonia (taking into account the components “lack of interest”, “lack of satisfaction”, and “work inhibition”) and lifetime suicidal ideations, recent suicidal ideations, and lifetime suicide attempts in a sample of physicians. More precisely, this relationship was studied taking into account the interpersonal psychological theory of suicide.

The discussion was organized into two parts. Firstly, the relationships between recent anhedonia or its three components (lack of satisfaction, loss of interest, and work inhibition) and the three components of suicide risk (lifetime suicidal ideations, recent suicidal ideations, and lifetime suicide attempts) will be discussed whilst taking into account the results or multivariate logistic regressions without the variables related to the interpersonal psychological theory of suicide (Thwarted Belongingness and Perceived Burdensomeness).

Secondly, the role of anhedonia in the interpersonal psychological theory of suicide will be examined taking into account the results of the mediation analyses as well as the results of the multivariate logistic regressions.

### Anhedonia and suicidal ideation

In the present study, recent anhedonia and especially the component of lack of satisfaction was significantly associated with lifetime and recent suicidal ideations. This significant result for lack of satisfaction was found when the other significant predictors were taken into account, in particular other depressive symptoms. We also found that the component “lack of interest in people” was not a significant predictor of lifetime suicidal ideations or recent suicidal ideations.

Several studies have reported significant association between anhedonia and suicidal ideation in various groups of subjects.

In psychiatric subjects, significant correlations were observed between the anhedonia-subscale of the BDI-II and suicidal ideation, as rated by the 9^th^ item of the BDI-II [18]. Significant correlations were reported for the lack of pleasure and lack of interest item of the BDI-II but not for the items measuring loss of energy (item 15) or loss of interest in sex (item 21) [18]. In healthy students, similar results were found using the SLIPS [19] with significant association between the SLIPS and suicidal ideation. The effect of depression was controlled for in both studies.

In subjects with anxious or depressive disorders, significant associations were found between the 9^Th^ item of the BDI-II and a subscale of the PAS rating trait-anticipatory anhedonia and the anhedonia subscale of the BDI. The correlations were significant for three items (lack of pleasure, lack of interest, lack of interest in sex) of the anhedonia subscale of the BDI-II [38].

Thus, in healthy and psychiatric subjects, suicidal ideations are associated with recent anticipatory and consummatory anhedonias, particularly in the social component, as well as trait-anticipatory physical anhedonia.

In our own sample of physicians, only the “lack of satisfaction” item of the BDI was associated with suicidal ideation, particularly when the other predictors (including depression) were controlled for. The results of the regression analyses were true not only for recent suicidal ideations but also for lifetime suicidal ideations.

Thus, recent anticipatory and consummatory anhedonia centered on the participant’s dissatisfaction as assessed by the lack of satisfaction item of the BDI-13 seemed to characterize physicians.

Contrary to healthy students or psychiatric subjects, the risk of suicidal ideations in physicians could be related solely to a lack of satisfaction and not to a double effect of lack of satisfaction and loss of interest in people.

Physicians’ dissatisfaction has increased during the last decades because of changes to the practice of medicine with a steep increase of administrative responsibilities and decreased autonomy [3]. Recently, one study [3] has proposed the development of the physicians’dissatisfaction scale which included 20 items and had satisfactory psychometric properties. Role uncertainty associated to loss of social esteem was the most dissatisfying factor. Burnout and depression are both consequences of physicians’ feelings of dissatisfaction [39]. One study reported that, in physicians, two essential dimensions of burnout (emotional exhaustion and depersonalization) were strongly related to self-perceived “insufficient” work ability [40]. Physicians’ burnout rates range from 30 to 65% across medical specialties [3].

### Anhedonia and suicide attempts

In the present study, anhedonia was associated with suicide attempts. When the different items of the anhedonia scale of the BDI were taken into account, only the item rating “work inhibition” was significantly associated with suicide attempts. The association between the item rating “work inhibition” and suicide attempts was found in logistic regressions as well as in mediation analyses.

Among the four different predictors that have been found significant in univariate analyses (Lack of Satisfaction-BDI-13, Work Inhibition-BDI-13, Cognitive/affective symptoms of depression and thwarted belongingness) and that have been introduced in the logistic regression, only work inhibition was a significant predictor.

It is important to point out that item # 15 of the BDI which rates “work inhibition” or “low energy” (depending on which version of the BDI is being used) does not rate directly anhedonia but, rather, a consequence of anhedonia such as fatigue.

Three studies have reported that anhedonia was not associated with suicide attempts [12, 19, 38] and one study suggested that the high prevalence of anhedonia in suicide attempters could only be explained by depression [41]. However, two recent studies reported significant associations between suicide attempt and anhedonia [10, 42].

The discrepancies between the various studies could be explained by a number of factors. Firstly, anhedonia was not always rated by specific rating scales and, when specific rating scales were introduced, they measured different types of anhedonia. Secondly, recent anhedonia could be associated with higher suicide risk. Thirdly, when anhedonia is studied in suicide attempters it is necessary to control for the potential effect of depressive symptomatology.

### Anhedonia and the interpersonal psychological theory of suicide

Previous studies in psychiatric [18] or healthy subjects [19] found that recent anhedonia and, in particular, the lack of interest in people are associated with suicidal ideation but not suicide attempt. One study [18] suggested, firstly, that recent loss of interest in people could potentially be associated with thwarted belongingness and, secondly, that recent loss of interest in people may lead to social isolation which constitutes a risk factor for suicidal ideation. Other studies in physicians [24] have reported that perceived burdensomeness leads to suicidal ideation whereas thwarted belongingness leads to suicide attempt. Moreover, one study [26] in 167 individuals aged 55 or over reported significant correlations between the SLIPS and rating scales that measure perceived burdensomeness and thwarted belongingness.

Regarding the role of anhedonia in the interpersonal psychological theory of suicide, the present study found that recent consummatory and anticipatory anhedonias, as rated by the anhedonia subscale of the BDI-13, partially or completely mediated the relationships between perceived burdensomeness or thwarted belongingness and life-time suicidal ideation, life-time suicide attempts or recent suicidal ideation.

Mediation analyses helped us understand the relationship between anhedonia, suicide risk and perceived burdensomeness or thwarted belongingness.

Partial mediations by anhedonia were found for associations between perceived burdensomeness or thwarted belongingness and life-time suicidal ideations. Complete mediations by anhedonia were found for associations between perceived burdensomeness or thwarted belongingness and recent suicidal ideations.

When the various items of the anhedonia subscale of the BDI-13 were taken into account, the items measuring “lack of satisfaction” and “work inhibition” were partial mediators in the three or four mediation analyses examining the relationships between perceived burdensomeness or thwarted belongingness and lifetime suicidal ideations or recent suicidal ideations. However, the item measuring “lack of interest in people” was only a partial mediator of the relationship between life-time suicidal ideations and perceived burdensomeness.

Our findings confirmed Winer et al.’s [18] suggestion that a loss of interest in people could be associated with thwarted belongingness as the regression analyses were significant between life-time suicidal ideations or recent suicidal ideation (independent variables) and loss of interest (dependent variable). Moreover, loss of interest was not a significant mediator between thwarted belongingness and life-time or recent suicidal ideations or between perceived burdensomeness and recent suicidal ideations.

To the best of our knowledge no study has, as yet, studied the potential role of anhedonia as a mediator of the relationships between suicide risk (suicidal ideation or suicide attempts) and thwarted belongingness or perceived burdensomeness.

Only one recent study by Golding et al.’s [26] with 167 subjects aged 55 and over, with no psychiatric disorders and recruited from two independent mTurk data collections in the United States of America, explored the relationships between insomnia symptoms, nightmares, and suicide risk using the Interpersonal Psychological Theory on Suicide, controlling for anhedonia, rated by the SLIPS. Participants filled out several rating scales including the SLIPS (measuring recent lack of interest in people), the Interpersonal needs questionnaire, the acquired capability for suicide scale-fearlessness about death and the suicidal behaviors questionnaire. The suicidal behaviors questionnaire measured suicide risk levels including suicidal thoughts, ideation, threats and attempts. Suicide risk was associated with anhedonia, and anhedonia was associated with burdensomeness and belongingness.

This study [26] confirmed the association between recent consummatory and anticipatory social anhedonia and burdensomeness and belongingness. In this study [26], the potential effect of depression was not controlled for and suicide risk assessment did not allow the distinction between suicidal ideations and suicide attempts.

Contrary to the results of Golding et al. [26], lack of interest in people did not play an important role in the relationship between perceived burdensomeness or thwarted belongingness and suicidal ideations in the present study. Among the different mediation analyses only one showed that “lack of interest in people” was a significant partial mediator between life-time suicidal ideations and perceived burdensomeness.

Regarding suicide attempts, the mediation analyses showed firstly that anhedonia rated by the Anhedonia-BDI-13 and secondly that Work Inhibition-BDI-13 were significant mediators of the relationship between suicide attempts and Thwarted Belongingness. Moreover, there were complete mediations hereby showing that anhedonia or work inhibition explained the relationship between thwarted belongingness and suicide attempts in its entirety. As previously emphasized in the discussion, the item “work inhibition” is not a direct measure of anhedonia per se but, rather, a measure of the consequence of anhedonia or cognitive inhibition or other depressive symptoms (e.g;. anxiety, psychomotor retardation…) [43].

Comparatively to the results of Fink-Miller [24] which showed that perceived burdensomeness was a significant predictor of suicidal ideation and that thwarted belongingness predicted prior suicide attempt in physicians, the present study showed that perceived burdensomeness and thwarted belongingness were predictors of suicidal ideations (lifetime or recent) whereas thwarted belongingness was a predictor of life-time suicide attempts.

### Limitations

The study had a number of limitations. The first concern was selection bias. Participants were physicians in only one town of Belgium, limiting external validity. The generalizability of findings to other populations was limited. Moreover, the response rate of the web survey was only 31%. Perhaps, physicians who have experienced or are experiencing suicidality may have been hesitant to participate. The second limitation was the use of proxy scales for the measurement of perceived burdensomeness and thwarted belongingness as opposed to the Interpersonal needs questionnaire. Further, the two proxy scales have insufficient reliabilities (Cronbach’s alpha < 0.7) and the time frame was variable (current state, two last weeks or lifetime). Thirdly, the mediation effects, derived from cross-sectional design of the study, do not imply causation [44]. Lifetime suicidal ideations (lf-SI) and suicide attempts (lf-SA) are most likely temporally prior to the timeframe of symptom assessment; the relationships there are thus not temporal but anachronistic. In this context, current symptoms must be considered either as potential outcomes rather than potentially causal correlates of lf-SI or lf-SA, or they must be considered as markers of a propensity to those symptoms (anhedonia, perceived burdensomeness or thwarted belongingness) which are presumed to have occurred in the past as well. Fourthly, further studies may examine the roles of different aspects of anhedonia (e.g., consummatory versus anticipatory) using specific rating scales. In the present study “Work inhibition” item of the Anhedonia scale of the BDI was rather an indirect measure of anhedonia.

## Conclusion

In a large sample of physicians, recent consummatory and anticipatory anhedonias, as rated by the anhedonia subscale of the BDI, were associated with recent and lifetime suicidal ideation. This association has been recently confirmed by a meta-analysis [45].There was also a significant relationship between anhedonia and suicide attempts. Among the three items of the anhedonia scale only the “work inhibition” item of the anhedonia subscale of the BDI was significantly associated with suicide attempts. In contrast with findings observed in psychiatric patients and in healthy students, the relationships between anhedonia and suicidal ideations were essentially found for the component “lack of satisfaction” of the anhedonia subscale of the BDI.

However, anhedonia, and in particular, the “dissatisfaction” component mediated the relationships between perceived burdensomeness or thwarted belongingness and recent or lifetime suicidal ideation in physicians.
